# Syncope in a Patient with Takotsubo Syndrome: Additional Issues to Consider

**DOI:** 10.5811/cpcem.53978

**Published:** 2026-01-14

**Authors:** John E. Madias

**Affiliations:** Icahn School of Medicine at Mount Sinai, New York, New York. Elmhurst Hospital Center, Department of Cardiology, Elmhurst, New York

To the Editor:

Virella et al^1^ reported on a 66-year old woman who sustained a fall with injury consequent to syncope in the setting of takotsubo syndrome (TTS), triggered while she was participating in a Zumba dance class, with subsequent fast recovery, and discharged after four days’ hospitalization. The authors reported that point-of-care ultrasound (POCUS) revealed apical hypokinesis with thickened basal septum, left ventricular outflow obstruction (LVOTO), and systolic anterior motion of the mitral valve (SAM-MV), which they attributed to a Venturi effect. The authors should be congratulated for the management of their patient.

The objective of this letter is to draw the authors’ attention to some issues needing further consideration, to further enhance the value of their work and contribute to the follow-up care of their patient. Consequently, I would appreciate the authors’ response to the following comments/questions, in view of the inclusion of reference #5 in their report:2 1) Was there any intraventricular pressure gradient recorded during POCUS or subsequent conventional echocardiograms (CECHO), during hospitalization? 2) Did the systolic murmur1 persist until discharge, or at follow-up? 3) Did the patient have a post-discharge CECHO? 4) Does the patient have underlying “sigmoid septum”^2^–^5^ on POCUS or CECHO? 5) Does the patient have underlying latent/overt hypertrophic cardiomyopathy,^2^–^5^ in view of the fact that the patient “reported experiencing intermittent lightheadedness over the prior one to two weeks while walking, which resolved with rest”?^1^ 6) The authors should also consider an alternative to the “Venturi effect” mechanism for the LVOTO, the SAM-MV, and apical hypokinesis,^1^ described in detail in their reference #5.^2^ 7) According to the above, one could surmise that this patient suffered an “increased afterload/segmental left ventricular coronary mismatch”-based TTS, rather than the “neurohumoral TTS type,”^3^–^5^ described in reference #5.^2^ Indeed, we should even consider that the presented patient with “apical ballooning” did not have TTS, after all; let’s not forget that the pathophysiology of TTS is still elusive!
